# Toxoplasmosis and Horse Meat, France

**DOI:** 10.3201/eid1707.101642

**Published:** 2011-07

**Authors:** Christelle Pomares, Daniel Ajzenberg, Loïc Bornard, Gilles Bernardin, Lilia Hasseine, Marie-Laure Dardé, Pierre Marty

**Affiliations:** Author affiliations: Université de Nice–Sophia Antipolis–Inserm U895, Nice, France (C. Pomares, P. Marty);; Université de Limoges, Limoges, France (D. Ajzenberg, M.L. Dardé);; Centre Hospitalier Universitaire de Nice, Nice (L. Bornard, G. Bernardin, L. Hasseine)

**Keywords:** parasites, toxoplasmosis, Toxoplasma gondii, horse meat, atypical strain, foodborne infections, France, letter

**To the Editor:** *Toxoplasma gondii* parasites are obligate intracellular apicomplexans that can infect virtually all warm-blooded animals; felids are definitive hosts. The most common sources of human infection are ingestion of tissue cysts in undercooked meat or of food or water contaminated with oocysts shed by felids and transplacental transmission. Acquired toxoplasmosis in immunocompetent humans is frequently asymptomatic but is associated with cervical or occipital lymphadenopathy in ≈10% of patients. Severe or fatal outcomes for immunocompetent patients have been attributed to the virulence of specific *T*. *gondii* genotypes (*1*). We describe 3 cases of toxoplasmosis caused by atypical strains probably acquired by from ingestion of raw horse meat imported from Canada and Brazil.

Patient 1, a 74-year-old man, was hospitalized locally (Antibes-Juan Les Pins, southern France) in March 2009 for asthenia and persistent febrile bronchitis. His medical history included severe smoking-related chronic obstructive pulmonary disease and coronary artery disease. He received broad-spectrum antimicrobial drugs and methylprednisolone. On day 23 after admission, he was transferred to our teaching hospital in Nice because of clinical deterioration and persistent fever. Disseminated toxoplasmosis was diagnosed on the basis of serologic evidence of recent primary *T. gondii* parasite infection and quantitative PCR detection of high *Toxoplasma* DNA levels in peripheral blood. Despite specific antitoxoplasma therapy with sulfadiazine and pyrimethamine, he remained febrile, his respiratory function worsened, and he died on day 27.

Patient 2, a 24-year-old pregnant woman, was hospitalized in Draguignan, France, in December 2009 for full-term delivery. Three weeks earlier, routine serologic testing showed *T. gondii* parasite infection seroconversion. The newborn’s and mother’s ophthalmologic examinations were unremarkable. Congenital toxoplasmosis was diagnosed on the basis immunoglobulin M in the infant’s serum, positive quantitative PCR of samples from the placenta, and strain isolation after inoculation of mice with a placental preparation. Sulfadiazine and pyrimethamine were started.

We performed a retrospective epidemiologic investigation of an unusual case of toxoplasmosis that occurred in March 1991. Patient 3, a 21-year-old pregnant woman living in the Nice area, was treated with spiramycine because routine serologic testing had shown *T. gondii* parasite infection seroconversion at 22 weeks’ gestation. Amniocentesis showed *T. gondii* tachyzoites in amniotic fluid by microscopic examination. At 26 weeks’ gestation, the woman underwent termination of pregnancy for ultrasonography-detected fetal severe abnormalities. Fetal necropsy showed numerous cerebral, cardiac, and hepatic abscesses with *T. gondii* tachyzoites. A few days after pregnancy termination, the woman experienced cervical lymphadenopathy, which lasted 3 years. She reported having eaten raw horse meat regularly during her pregnancy.

Genetic analyses with microsatellite markers of the *Toxoplasma* spp. strains isolated from the 3 patients found 3 different atypical genotypes. Atypical strains are common in South America but unusual in France, where >95% of reported strains collected from human and animal toxoplasmosis cases belonged to the type II clonal lineage (*2**,**3*). Hence, isolation of an atypical *Toxoplasma* genotype from a patient in France strongly suggests contamination by a non-European strain, either during residence abroad or after ingestion of imported meat. Epidemiologic investigation of our case-patients that included questioning relatives, patients, and butchers found that eating raw horse meat imported from Canada (patient 1) or Brazil (patient 2) was the most likely source of the parasites. The geographic origin of the horse meat eaten by patient 3 is unknown.

Moreover, an atypical *T. gondii* strain was isolated after mouse inoculation with horse meat from the first patient’s butcher. In all 3 cases, close relatives encouraged the patients to eat raw horse meat regularly because the practice is traditionally thought to reinforce health. Human toxoplasmosis cases associated with horse meat consumption are rarely reported but are probably underestimated (*2*). In the European Union, France and Italy account for more than two thirds of all horse meat eaten, predominantly raw, thereby increasing the likelihood of infection by various parasites, including *Trichinella* spp. and *Toxoplasma* spp (*4*). Under natural conditions, serologic prevalence of *T. gondii* parasites in horses worldwide may range from 0% to 80% (*5*). Many factors could account for this variation, including the sensitivity and specificity of the serologic test, ages of animals, geographic area and hygienic condition of farm management (*5*). The only prevalence survey of horses slaughtered for food that we are aware of was conducted in Canada and the United States and found antibodies to *T. gondii* parasites in 124 (6.9%) of 1,788 serum samples (*6*).

*T. gondii* tissue cysts in meat are immediately killed by reaching an internal temperature of 67°C in all parts of meat during cooking (*7*). Deep freezing (<–12°C for at least 3 days) of meat before cooking is recommended because it reduces the risk for infection by inactivating most tissue cysts (*7*). These precautions are often not applied to horse meat because these imported carcasses are usually shipped as “fresh meat” and frequently eaten raw. Eating raw horse meat imported from non-European countries may expose consumers to high inocula of highly virulent atypical *Toxoplasma* spp. strains, which may cause life-threatening primary infection (case-patient 1) or severe congenital toxoplasmosis with atypical outcome of acquired toxoplasmosis in the mother (case-patient 3). Risk assessment for toxoplasmosis from horses slaughtered for food and imported into the European Union, as was recently done in France for ovine meat, is urgently needed (*3*).
